# Below-ground tissues of *Capsicum annuum* respond to conserved bacterial peptides with pattern-triggered immunity

**DOI:** 10.17912/micropub.biology.001726

**Published:** 2025-08-08

**Authors:** Morgan Murff, Brooke Pilkey, Rebecca Leuschen-Kohl, Anjali Iyer-Pascuzzi

**Affiliations:** 1 Department of Botany and Plant Pathology, Purdue University West Lafayette, West Lafayette, Indiana, United States

## Abstract

Plants have an innate immune system that deters and reduces infection by pathogenic microbes. *Solanaceous* plants such as *Solanum lycopersicum* (tomato) use cell-surface immune receptors to perceive microbe-associated molecular patterns (MAMPs) and activate pattern-triggered immunity (PTI), but these responses have been seldom explored in roots of other members of the *Solanaceae* . To investigate the PTI responses in roots of *Capsicum annuum* (pepper), oxidative luminescence and temporary root growth inhibition assays were used to measure PTI upon treatment with three bacterial MAMPs: two flagellin (flg)-derived peptides (flg22 and flgII-28), and one cold shock protein-derived peptide (csp22), in multiple pepper accessions. Our results show that pepper roots exhibit a significant increase in ROS production in response to csp22, flg22, and flgII-28 treatment, while only flg22 causes temporary root growth inhibition. PTI responses differ in amplitude among MAMPs and genotypes. Together, these results suggest that downstream immune signaling or immune receptor expression may differ among pepper genotypes and MAMP treatments and highlight the importance of investigating immune response variation in various crop plants.

**Figure 1. Pepper roots have genotype and MAMP-specific PTI responses:**
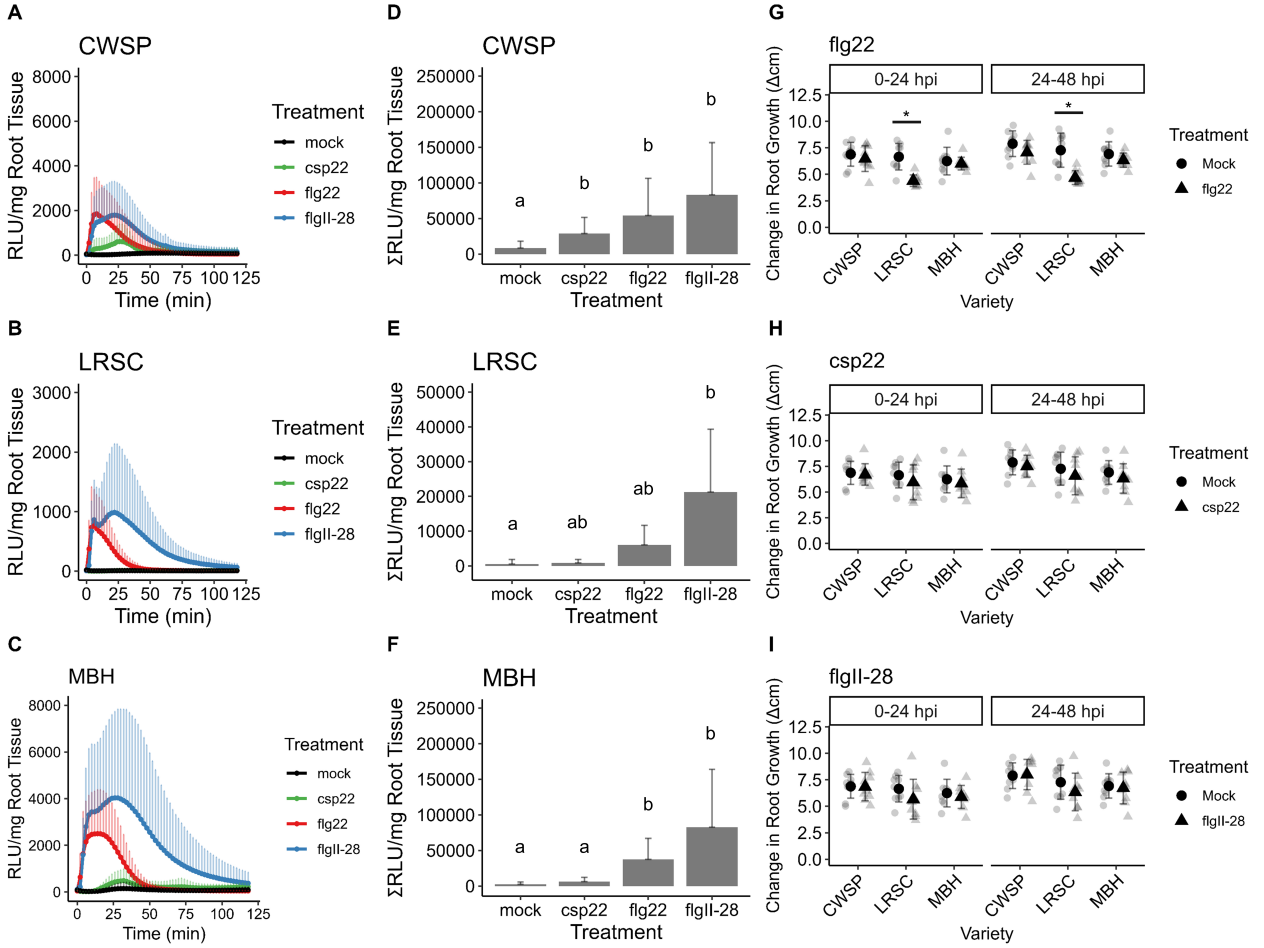
**[A-C]** ROS burst measured in relative light units (RLU) /mg root tissue over time in response to 1 µM csp22, flg22, flgII-28 or mock (water) for 15-day-old seedlings of **[A]** California Wonder Sweet Pepper (CWSP), **[B]** Long Red Slim Cayenne (LRSC), and **[C]** Mexibell Hybrid (MBH) accessions (n=6 per treatment). **[D-F]** Area under the ROS burst curve measured as the average sum of RLU/min in response to 1 µM csp22, flg22, and flgII-28 or mock (water) in **[D]** CWSP, **[E]** LRSC, and **[F]** MBH. Temporary root growth inhibition measured as the change in root growth (cm) 0-24 hours post inoculation (hpi) and 24-48 hpi after treatment with **[G]** 1 µM flg22 or mock(water), **[H]** 1 µM csp22 or mock (water), or **[I]** 1 µM flgII-28 or mock (water). 1D-1F: ANOVA, Tukey HSD (p < 0.05); 1G-I: T-Test (* p < 0.01)

## Description

Plants possess an innate immune system that plays a critical role in preventing and deterring pathogens. This immune response is composed of two primary layers of defense: pattern-triggered immunity (PTI) and effector-triggered immunity (ETI) (Yu et al., 2024). In PTI, microbe-associated molecular patterns (MAMPs) are recognized by pattern recognition receptors (PRRs) at the cell surface and initiate downstream defense responses such as reactive oxygen species (ROS) burst, calcium signaling, root growth inhibition, and actin filament rearrangement (Naveed et al., 2020; Zhang et al., 2025; Shu et al., 2023). These responses can lead to broad-spectrum resistance to pathogens, and applications of these processes have been successful in increasing crop resilience to a range of phytopathogenic bacteria (Lacombe et al., 2010).

Soilborne pathogens are a major cause of plant disease, and root immune responses are critical for alleviating crop loss. Although the basic tenets of PTI are similar in above and belowground organs and across species, PTI responses show noticeable distinctions within species, tissues, and cell types (Beck et al., 2014; Chuberre et al., 2018, Leuschen-Kohl et al. 2024). For example, in tomato roots, PTI responses depend on the developmental stage of the root tissue (Leuschen-Kohl et al., 2024). Within the *Solanaceace* , potato ( *Solanum tuberosum* ) is responsive to flgII-28 and initiates [Ca ^2+^ ] elevation, ROS production, MAPK phosphorylation, and reprogramming of defense genes (Moroz and Tanaka 2020) while eggplant ( *S. melongena* ) does not (Wei et al., 2016). Additionally, flgII-28 treatment in leaves activates tomato homologs of Arabidopsis MPK3/6 but only MPK6 in potato (Leuschen-Kohl et al., 2014; Moroz et al., 2019). We hypothesized that similar variations in response to MAMPs will be found in roots of other *Solanaceous* species. Here, we examined accession-specific responses to PTI in *Capsicum* *annuum* using oxidative burst luminescence analyses and root growth inhibition assays.

We first examined ROS burst in roots of three genotypes of *C. annuum* . A ROS burst in response to MAMPs is a hallmark of PTI, but genotype- and MAMP-specific differences have been found in *Solanum* *lycopersicum* roots (Leuschen-Kohl et al. 2024). To test this in peppers, whole roots from three different *C. annuum* genotypes, California Wonder Sweet Pepper (CWSP), Mexibell Hybrid (MBH), and Long Red Slim Cayenne (LRSC) were each treated with 1 µM of each MAMP (flg22, csp22, or flgII-28). CWSP responded to all three elicitors, while MBH and LRSC only responded to flg22 and flgII-28 ( Figure 1A- C). For both LSRC and MBH, the temporal dynamics differed between MAMPs. The ROS burst due to flg22 was much shorter and total ROS over time (120 min) was not significantly different than mock in LRSC ( Figure 1B, E). Csp22 elicited a significant response in CWSP, but not LSRC or MBH, suggesting that these cultivars either do not express the CORE receptor or csp22 is weakly recognized in these plants ( Figure 1A- F). Previous work found that CWSP did not respond to csp22 in leaves of 5-week-old plants (Wei et al. 2018), suggesting that expression of the CORE receptor in pepper differs between above and below-ground plant tissues or decreases with age.

Roots respond to both long-term and short-term exposure to MAMPs by inhibiting their growth (Poncini et al. 2017), although temporary root growth inhibition (tRGI) appears to vary by MAMP treatment (Leuschen-Kohl et al. 2024). To assess this in peppers, roots from CWSP, MBH, LRSC were treated with bacterial MAMPs (1 µM flg22, csp22, or flgII-28) and root growth was measured over 48 hours. flg22, flgII-28, and csp22 treatments did not lead to a significant decrease in root growth at 0-24 hpi or 24-48 hpi in MBH and CWSP ( **Figure 1G** ). LRSC displayed a significant early root growth inhibition in response to flg22 at 0-24 hpi and 24-48 hpi, but not in response to other MAMPs. Thus for most PRR-MAMP combinations, the MAMP elicited a ROS burst but did not elicit tRGI. This suggests that, like tomato, tRGI and ROS burst are uncoupled processes in pepper for most PRR-MAMP combinations.

Together, our results reveal that pepper root immune responses to MAMPs are genotype and elicitor-specific and show distinct patterns among MAMPs. This could be due to differences in signaling downstream of PRRs or variations in immune receptor expression among pepper cultivars. Understanding genetic variation in response to MAMPs is important for developing sustainable strategies for disease control.

## Methods

Seeds of pepper ( *C. annuum* ) accessions (listed below) seeds were sterilized for 5 minutes in 50% bleach + 0.001% Tween, washed with 70% ethanol for 30 seconds, and rinsed three times with pure water. Seeds were plated on 1% water agar (WA) plates, placed in 4 °C overnight, and transferred to room temperature (22°C) with 16:8 day/night cycle.

For RGI analysis, fifteen-day-old seedlings (about 4-5cm in length) were transferred to fresh plates, scanned, and incubated for 24 hours in the conditions above. After 24 hours, seedling roots were treated with 200µL of elicitor (1µM flg22, 1µM flgII-28, 1µM csp22, or water). All elicitors were dissolved in water. The seedlings were then scanned at 24- and 48-hours post-treatment, and root length was quantified with ImageJ.

The ROS assay was performed as published by Leuschen-Kohl et al. 2024 with modifications. Briefly, fifteen-day-old seedlings were cut at the root-shoot junction. Root samples were weighed, placed in a 96-well plate (Perkin Elmer, OptiPlate-96) with 200µL of pure water, then left overnight in the dark. The next day, the water was replaced with 200µL of the master mix for each peptide elicitor. The master mix was composed of 1.5X L-012 and 1.5X HPSS and 1µM of the corresponding peptide elicitor. An Infinite 200 Pro Luminescent Microplate Reader (Tecan Life Sciences, Switzerland) was used to measure relative light units (RLU) for 60 minutes. Three biological replicates were used for each analysis, with 5-6 roots per treatment. The data was normalized and expressed as RLU per milligram of fresh weight.

Statistical analyses were conducted in R version 3.4.0. Data distribution was assessed, and tests appropriate to the data distribution were applied.

## Reagents

**Table:** 

**Accession**	**Name**	**Source**
*Capsicum annuum* L. cv. California Wonder	California Wonder Sweet Pepper (CWSP)	OSC seeds
*Capsicum annuum* L. cv. MexiBell Hybrid	Mexibell Hybrid (MBH)	OSC seeds
*Capsicum annuum* L. cv. Long Red Cayenne Pepper	Long Red Slim Cayenne (LRSC)	OSC seeds
**Bacterial Elicitor**	**Amino Acid Sequence**	**Source**
flg22 ^Pst^	QRLSTGLRVNSAQDDSAAYAAS Purity: 98.98%	EZBiolabs
csp22 ^Rsol^	ATGTVKWFNETKGFGFITPDGG Purity: 98.13%	EZBiolabs
flgII-28 ^Pst^	ESTNILQRMRELAVQSRNDSNSATDREA Purity: 95.4%	GenScript
**Molecular Tool**	**Name**	**Source**
HPSS	Horseradish peroxidase stock solution	Thermo Fisher Scientific
LSS	L-012 stock solution	Wako Chemicals USA
